# Comparative Genomics Analysis of *Streptococcus tigurinus* Strains Identifies Genetic Elements Specifically and Uniquely Present in Highly Virulent Strains

**DOI:** 10.1371/journal.pone.0160554

**Published:** 2016-08-09

**Authors:** Seydina M. Diene, Patrice François, Andrea Zbinden, José Manuel Entenza, Grégory Resch

**Affiliations:** 1 Genomic Research Laboratory, Geneva University Hospitals, Geneva, Switzerland; 2 Institute of Medical Virology, University of Zurich, Zurich, Switzerland; 3 Department of Fundamental Microbiology, University of Lausanne, Lausanne, Switzerland; ContraFect Corporation, UNITED STATES

## Abstract

*Streptococcus tigurinus* is responsible for severe invasive infections such as infective endocarditis, spondylodiscitis and meningitis. As described, *S*. *tigurinus* isolates AZ_3a^T^ and AZ_14 were highly virulent (HV phenotype) in an experimental model of infective endocarditis and showed enhanced adherence and invasion of human endothelial cells when compared to low virulent *S*. *tigurinus* isolate AZ_8 (LV phenotype). Here, we sought whether genetic determinants could explain the higher virulence of AZ_3a^T^ and AZ_14 isolates. Several genetic determinants specific to the HV strains were identified through extensive comparative genomics amongst which some were thought to be highly relevant for the observed HV phenotype. These included i) an iron uptake and metabolism operon, ii) an ascorbate assimilation operon, iii) a newly acquired PI-2-like pilus islets described for the first time in *S*. *tigurinus*, iv) a hyaluronate metabolism operon, v) an Entner-Doudoroff pathway of carbohydrates metabolism, and vi) an alternate pathways for indole biosynthesis. We believe that the identified genomic features could largely explain the phenotype of high infectivity of the two HV *S*. *tigurinus* strains. Indeed, these features include determinants that could be involved at different stages of the disease such as survival of *S*. *tigurinus* in blood (iron uptake and ascorbate metabolism operons), initial attachment of bacterial pathogen to the damaged cardiac tissue and/or vegetation that formed on site (PI-2-like pilus islets), tissue invasion (hyaluronate operon and Entner-Doudoroff pathway) and regulation of pathogenicity (indole biosynthesis pathway).

## Introduction

*Streptococcus tigurinus* is a recently identified species belonging to the *Streptococcus mitis* group. It is a commensal of the human oral cavity [1, 2] and can be responsible of severe invasive infections such as infective endocarditis, spondylodiscitis and meningitis [3–9]. Compared to other viridans streptococci, *S*. *tigurinus* might currently be underreported due to limited identification in a clinical routine laboratory [3]. Indeed, conventional phenotypic methods do not provide accurate identification of *S*. *tigurinus* because of the morphological resemblance to its closest related species, i.e., *S*. *mitis*, *Streptococcus oralis*, *Streptococcus pneumoniae*, *Streptococcus pseudopneumoniae* and *Streptococcus infantis*. Analysis of the 5’-end of the 16S rRNA gene is mandatory for accurate identification of *S*. *tigurinus* since a significant sequence demarcation was demonstrated [1, 4].

*S*. *tigurinus* was found to be highly virulent in our rat model of experimental endocarditis [10]. Its capacity to induce infective endocarditis was similar to that of *Staphylococcus aureus* or enterococci but important variability was observed in the virulence capacity of different *S*. *tigurinus* strains [10]. When injected at similar inoculum sizes, the highly virulent (HV) strains AZ_3a^T^ and AZ_14 induced infective endocarditis in ≥80% of the rats, compared to the low virulent (LV) *S*. *tigurinus* isolate AZ_8 which produced infective endocarditis in only 56% of the animals [10]. Moreover, these phenotypes correlated with enhanced capabilities of the HV strains to adhere to and invade endothelial cells [10]. A well-studied strain 859 showing similar infectivity rate as *S*. *tigurinus* AZ_8 in the experimental infective endocarditis model was previously classified as *S*. *mitis* and turned out as *S*. *tigurinus* after 16S rRNA gene analysis [10].

In the present study, we performed de novo whole-genome sequencing of four *S*. *tigurinus* strains and performed comparative genomics analyses including additional previously sequenced *S*. *tigurinus* strains to seek whether genetic differences could potentially explain differences observed in strain’s virulence phenotypes.

## Materials and Methods

### Bacterial strains

*S*. *tigurinus* isolates AZ_3a^T^ (CCOS 600^T^, Culture Collection of Switzerland), AZ_8 (CCOS 678) and AZ_14 (CCOS 689) are clinical strains recovered from blood samples from patients diagnosed with infective endocarditis [3]. *S*. *tigurinus* strain 859 was isolated from the nasopharynx of a children from South Africa [11]. In order to analyse a non-invasive *S*. *tigurinus* strain by comparative genomics, the *S*. *tigurinus* strain ATCC15914 originally isolated from human throat [4], was included. Additional *S*. *tigurinus* strains 1366 (parental wild type strain, isolated from the preoperative joint aspirate), 2425 and 2426 (isogenic small-colony variants of strain 1366, isolated from periprosthetic tissue biopsy) were previously sequenced and were originally isolated from a patient with prosthetic joint infection [5, 12]. Bacterial stocks were kept frozen at -80°C in 20% glycerol and grown on Columbia agar plates containing 5% defibrinated sheep blood (bioMérieux, Marcy l’Etoile, France) at 37°C with CO_2_ for 24 h.

### Genome sequencing, assembly and annotation

Genomic DNA from *S*. *tigurinus* strains AZ_8, AZ_14, ATCC15914 and 859 were purified using a Wizard Genomic DNA purification kit (Promega, Dübendorf, Switzerland). Genomic DNA libraries from AZ_8, AZ_14, and 859 were prepared using 1μg of the purified genomic DNA and the TruSeq DNA LT Sample Preparation Kit according to the manufacturer’s protocol (Illumina, San Diego, USA; Cat. No. FC-121-2001). The resulting libraries were pooled into a single library for paired-end sequencing of 2x100-bp on the Illumina HiSeq 2500 using TruSeq PE Cluster Kit v3 (Cat. No. PE-401-3001) and TruSeq SBS Kit v3 (Cat. No. FC-401-3001). Data were processed using the Illumina Pipeline Software package v1.82 and aligned using Eland v2e. Assembly of paired-end reads was done with Edena [13]. ATCC15914 isolate was sequenced using the PacBio (Pacific Biosciences) technology [14]. Contigs were submitted to NCBI for automated annotation and publication in databases. Assembled contigs of strains AZ_3a^T^, 1366, 2425, 2426 or full genome of *Streptococcus oralis* strain Uo5 (closest relative of *S*. *tigurinus*) and full annotations were obtained from NCBI (accession numbers: AORU01, AORX01, ASWZ01, ASXA01, 331265438 respectively). Full genome of *Streptococcus oralis* strain Uo5, which is the closest relative of *S*. *tigurinus*, was obtained from NCBI (accession number 331265438).

### Availability of data and materials

The genome sequences of the four *S*. *tigurinus* strains *de novo* sequenced in this study have been deposited in the DDBJ/EMBL/GenBank database under the following accession numbers: *S*. *tigurinus* AZ_8, LNVF00000000; *S*. *tigurinus* AZ_14, LNVG00000000; *S*. *tigurinus* 859, LNVH00000000; and *S*. *tigurinus* ATCC15914, PRJNA302887.

### Phylogenetic analysis

Fasta files of Open Reading Frames (ORFs) (both nucleotide and amino-acids sequences) from each genome were downloaded from NCBI and used by two in-house perl scripts (available upon request) to build a phylogenetic tree. Briefly, core proteins, i.e. proteins encoded on the genome of the eight *S*. *tigurinus* strains and *S*. *oralis* Uo5, were identified by blasting each single protein sequence of a given strain to each single protein sequence of the eight other strains using BlastP with e-value threshold of 10^−10^. Corresponding nucleotide sequences of genes coding for core proteins present in single copies in all genomes were further used to build a multiple alignment with MAFFT-7.187 [15]. Newick formula was obtained by submitting the MAFFT output file to FastTree-2.1.7 [16]. Finally, the Newick formula was uploaded in Njplot 2.3 (http://doua.prabi.fr/software/njplot) [17] to visualize and root the corresponding phylogenetic tree using *S*. *oralis* Uo5 as outgroup. This tree was named Tree_9strains. A similar approach was used to generate a tree for the three strains of interest only, namely AZ_3a^T^, AZ_14 and AZ_8. In this case, the tree was rooted using AZ_8 as outgroup and was named Tree_3 strains.

### Comparative genomics analyses

The core proteome comparison of all strains was performed using the described script “get_homologues.pl” [18]. In this analysis, the core proteome of each strain was identified and then the pairwise average similarity was determined. The genome comparison and the circular representation of this analysis were performed using the described CGView Comparison Tool program [19]. To investigate in details the genetic differences between HV and LV strains, Count software [20] was used with Tree_3strains and the corresponding matrix of absence/presence of genes for each strain generated by the first perl script and supplemented with annotations obtained from NCBI. At first, the Family history by Dollo Parsimony tool was used to identify genes that were acquired and maintained specifically in both HV strains and absent from the LV strain. Using a similar approach with Tree_9strains, the presence of homologs to genes identified in the first analysis was checked in all other strains included in this study. In addition, contig sequences of each strain were submitted to RAST v 2.0 [21]. Topology of LPXTG proteins was predicted using Phobius (http://phobius.sbc.su.se) and Clustal Omega was used to perform alignment of gene products (http://www.ebi.ac.uk/Tools/msa/clustalo).

## Results

### Whole-genome sequencing, assembly and annotation

More than 12 millions high quality 100-bp paired-end reads were obtained for each of the four strains that were *de novo* sequenced in this study and no trimming was therefore needed. Reads assembled into 35, 38, and 14 contigs for strains AZ_8, AZ_14, and 859 respectively (Table 1). PacBio sequencing of strain ATCC15914 resulted in full genome recovery on a single contig. No plasmid sequences were identified in the assembled contigs. The whole genome sequences of *S*. *tigurinus* AZ_3a^T^, 1366, 2425, 2426 and *S*. *oralis* Uo5 were retrieved from NCBI database and consisted in 22, 14, 15, 25, and 1 contigs, respectively (Table 1). Genome sizes of all strains ranged from 1.87 Mb to 2.18 Mb. Number of ORFs automatically predicted by RAST server ranged from 1’842 to 2’191 encoding between 1’833 and 2’141 proteins (Table 1). %GC content of all strains ranged from 39.17% to 45.48% and, interestingly, the genomes of the two HV strains harbored the lowest %GC contents (39.17% and 39.9% for AZ_14 and AZ_3a^T^, respectively).

**Table 1:** General features of genomes investigated in this study.

Strain	Isolation source	Accession number	Number of contigs	Genome size (Mb)	% GC content	Number of genes	Number of proteins
***Sequenced genomes in this study***							
*S*. *tigurinus*	Infection	LNVF00000000	35	2.13	42.20	2’134	2’079	
AZ_8	(blood)							
*S*. *tigurinus*	Infection	LNVG00000000	38	1.97	39.17	2’142	1’893
AZ_14	(blood)						
*S*. *tigurinus*	Carriage	PJRNA302887	1	1.91	41.40	1’918	1’845
ATCC15914	(throat)						
*S*. *tigurinus*	Carriage	LNVH00000000	14	2.04	40.46	2’066	2’010
859	(nasopharynx)						
***Genome sequences retrieved from NCBI database***							
*S*. *tigurinus*	Infection	AORU01	22	2.18	39.98	2’191	2’141
AZ_3a^T^	(blood)						
*S*. *tigurinus*	Infection	AORX01	14	1.87	45.34	1’891	1’833
1366	(knee joint fluid)						
*S*. *tigurinus*	Infection	ASWZ01	15	1.87	45.48	1’903	1’833
2425	(knee tissue biopsy)						
*S*. *tigurinus*	Infection	ASXA01	25	1.88	41.51	1’842	1’842
2426	(knee tissue biopsy)						
*S*. *oralis*	Carriage	331265438	1	1.96	41.10	1’991	1’909
Uo5	(mouth)						

### Phylogeny on core genes

**Fig 1:**
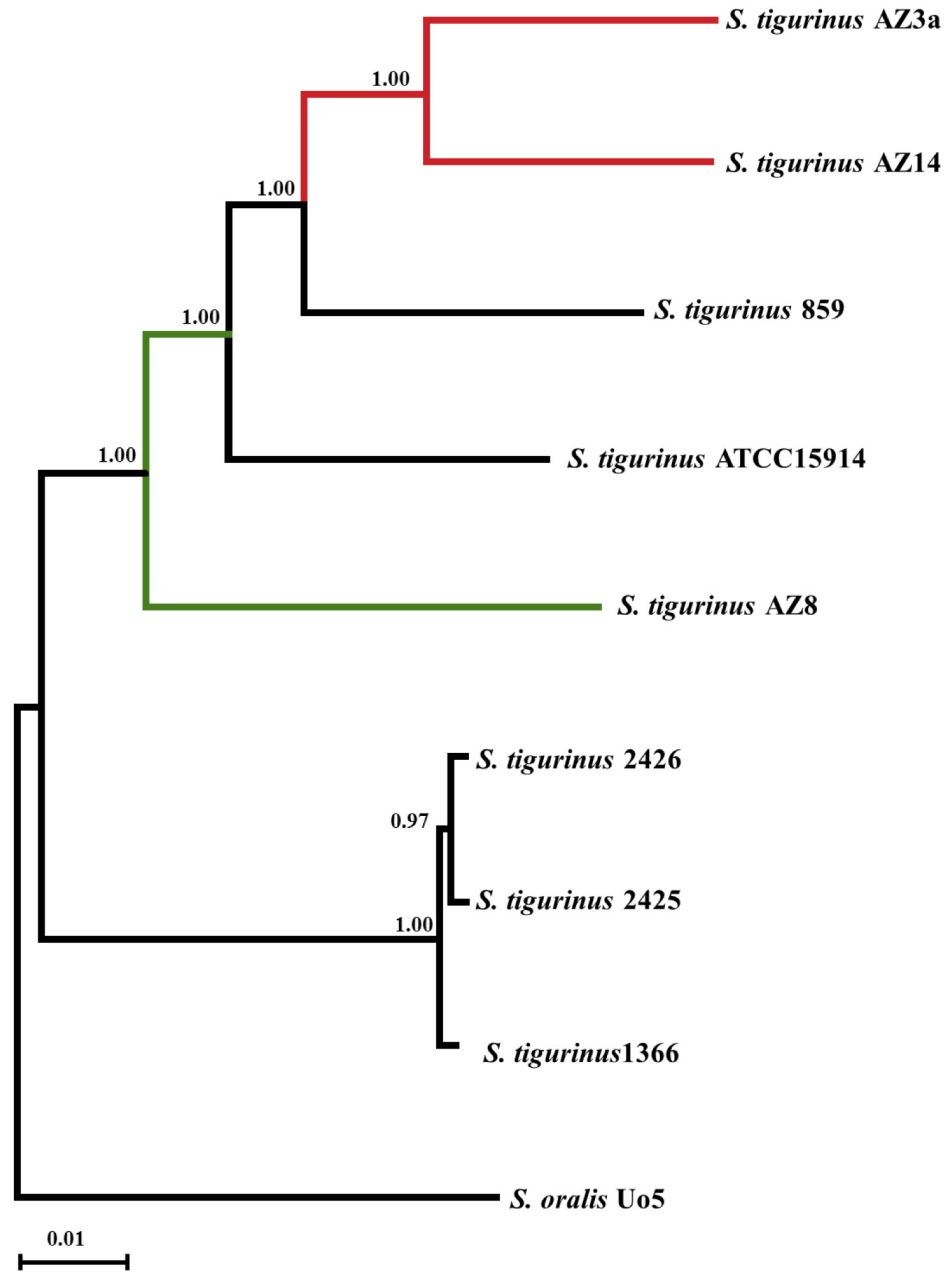
Phylogenetic rooted tree based on 1'331 single copy core genes. Both HV strains cluster together (AZ_3a^T^ and AZ_14, red branches) far from the LV strain (AZ_8, green branch). *S*. *oralis* Uo5 was chosen as outgroup.

The phylogenetic tree obtained from the alignment of 1’331 core genes present in single copies in the core genome of the eight *S*. *tigurinus* strains and *S*. *oralis* Uo5 is presented in Fig 1. As expected, *S*. *oralis* Uo5 (*S*. *tigurinus* most closely relative) used as outgroup readily diverged from the *S*. *tigurinus* strains. Of note, clustering of strains 2425 and 2426 together with strain 1366 supported accuracy of the phylogenetic tree. Indeed, strains 2425 and 2426 were previously described as two highly similar small-colony variants derived from the parental wild-type strain 1366 [12]. Interestingly, the two HV strains AZ_3a^T^ and AZ_14 clustered together (red branches, Fig 1) far from the LV strain AZ_8 (green branch, Fig 1).

### Pairwise comparison of the core proteomes

**Fig 2:**
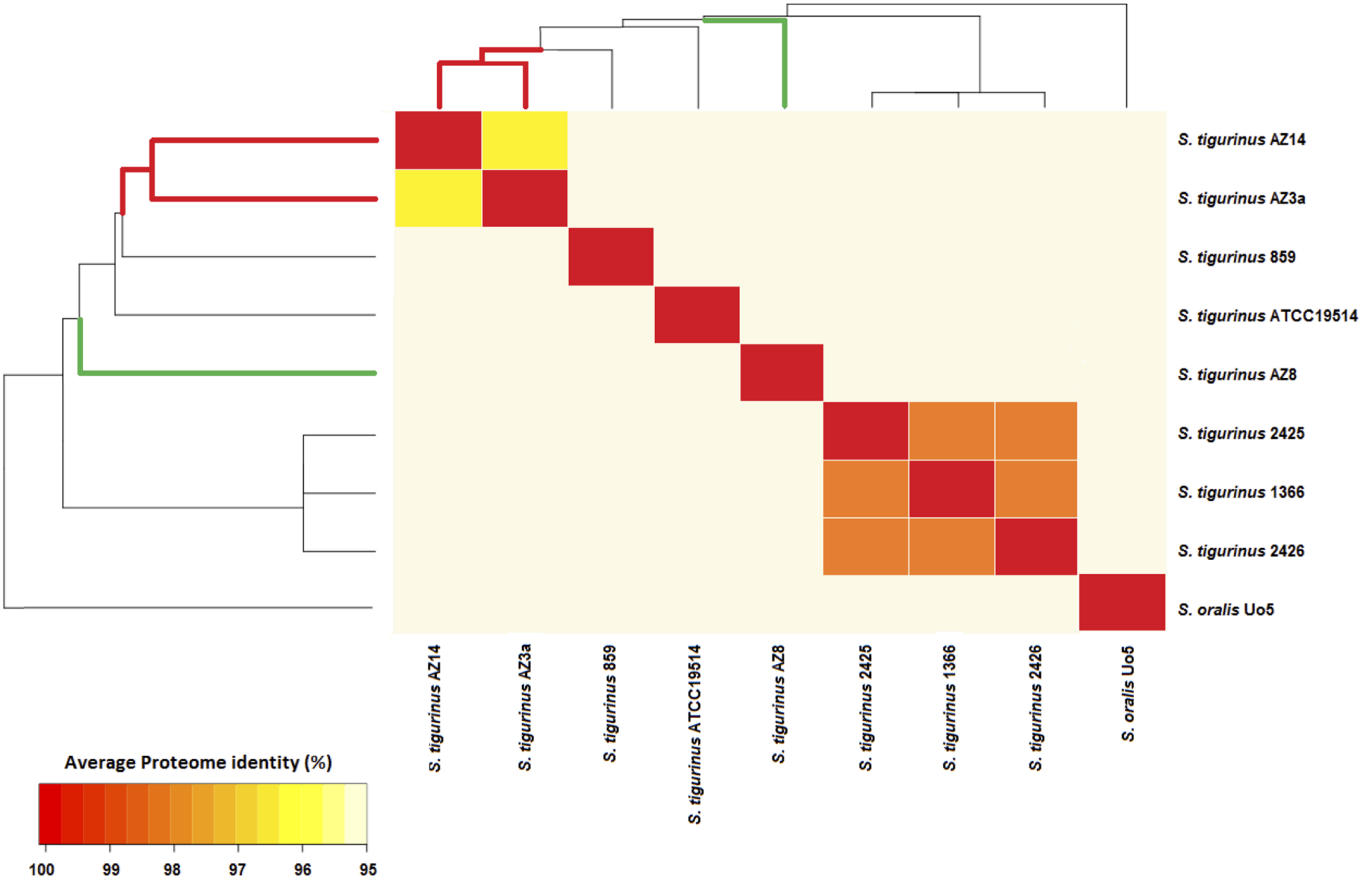
Pairwise comparison based on 1'331 ORFs belonging to the core proteome.

In addition to the phylogenetic analysis from the multiple alignment of 1’331 single copies core genes (see above), the corresponding core proteomes were compared. As represented in Fig 2, the average % of similarity between the core proteomes of each pair of strains ranged from 94.9% (between *S*. *oralis* Uo5 and AZ_3a^T^ or AZ_8) to 98.8% (between the three isogenic strains 1366, 2425 and 2426). Similar to the observation made in the phylogenetic tree (see above), high homology between strains 1366, 2425, 2426 (98.8%; highlighted in orange, Fig 2) supported accuracy of the core proteome comparative analysis. Interestingly, besides this cluster of isogenic strains, the core proteomes of the two HV strains AZ_3a^T^ and AZ_14 exhibited the highest % of identity (96.3%; highlighted in yellow, Fig 2).

### Genes specifically acquired in both HV strains

After having identified some general trends specific to the HV strains through phylogenetic analysis using core genes and pairwise comparison of core proteomes we decided to focus on and investigate further genetic determinants acquired and/or maintained in both HV strains but absent from the LV strain. Indeed we thought that such determinants could represent good candidates to explain, at least in part, the observed phenotype of increased infectivity of HV strains in the experimental infective endocarditis model [10]. Comparison of both HV strains with the LV strain using the Family history by Dollo Parsimony tool of Count software revealed 188 genes found in both HV strains that were absent in the LV strain (S1 Table). Amongst the list, several gene clusters harboring significant numbers of annotated gene products (S1 Table, highlighted in light grey)—i.e. having known potential functions, in the opposite of many other gene products annotated as hypothetical proteins—are further described thereafter.

### Carbohydrates metabolism

**Hyaluronate utilization:** A cluster of 13 genes involved in hyaluronate utilization was identified in the two HV strains (*AZ3a_ORF_19305* to *AZ3a_ORF_19365* and *AZ14_ORF_08215* to *AZ14_ORF_08155* in AZ_3a^T^ and AZ_14, respectively, Table 2 and S1 Fig). This operon was absent from *S*. *oralis* Uo5 and all other *S*. *tigurinus* strains studied except ATCC15914 (Table 2). Genes coding for enzymes involved in substrate degradation and transformation such as hyaluronate and oligohyaluronate lyases (encoded by *hylA* and *ohl*, respectively), unsaturated glucuronyl hydrolase (*ugl*), a deshydrogenase (*kduD*), an isomerase (*kduI*), hyaluronate-oligosaccharide-specific phosphotransferase system components (*PTSa-d*) and a repressor of the PTS system (*regR)* were present in this gene cluster. A very similar cluster was found in *S*. *pneumoniae* TIGR4 (S1 Fig).

**Table 2:** Presence and absence of the identified relevant gene clusters in the genomes of all strains investigated in this study. A black box means a homolog to the corresponding gene found in AZ_3a^T^ is present on the genome of the considered strain; a white box means no homolog to the corresponding gene found in AZ_3a^T^ is present on the genome of the considered strain.

ORF N°	Gene name	AZ_8 (LV)	AZ_3a^T^ (HV)	AZ_14 (HV)	ATCC 15914	1366	2425	2426	859	Uo5
AZ3a_ORF_0110920	*pitA*									
AZ3a_ORF_11620	*sipA*									
AZ3a_ORF_11625	*pitB*									
AZ3a_ORF_11630	*srtG1*									
AZ3a_ORF_11635	*srtG2*									
										
AZ3a_ORF_13870	*trpAa*									
AZ3a_ORF_13865	*trpAb*									
AZ3a_ORF_13840	*trpEa*									
AZ3a_ORF_13845	*trpEb*									
AZ3a_ORF_13860	*trpB*	**truncated**								
AZ3a_ORF_13850	*trpC*									
AZ3a_ORF_13855	*trpD*									
										
AZ3a_ORF_18870	*pitC*									
AZ3a_ORF_18875	*pitD*									
AZ3a_ORF_18880	*pitA*									
AZ3a_ORF_18890	*reg_SK*									
AZ3a_ORF_22340	*reg_RR*									
										
AZ3a_ORF_19305	*hylA*									
AZ3a_ORF_19310	*kdgA*									
AZ3a_ORF_19315	*kdgK*									
AZ3a_ORF_19320	*kduI*									
AZ3a_ORF_19325	*kduD*									
AZ3a_ORF_19330	*PTSa*									
AZ3a_ORF_19335	*ugl*									
AZ3a_ORF_19340	*PTSb*									
AZ3a_ORF_19345	*PTSc*									
AZ3a_ORF_19350	*PTSd*									
AZ3a_ORF_19355										
AZ3a_ORF_19360	*ohl*									
AZ3a_ORF_19365	*regR*									
										
AZ3a_ORF_21750	*ulaG*									
AZ3a_ORF_21755	*ulaR*									
AZ3a_ORF_21760	*sgaESgbE*									
AZ3a_ORF_21765	*sgaU*									
AZ3a_ORF_21770	*sgaH*									
AZ3a_ORF_21775	*ulaA*									
AZ3a_ORF_21780	*ulaB*									
AZ3a_ORF_21785	*ulaC*									

**Entner-Doudoroff pathway:** Both HV strains and ATCC15914 harbored genes *kdgA* (*AZ3a_ORF_19310* and *AZ14_ORF_08210* in AZ_3a^T^ and AZ_14, respectively) and *kdgK* (*AZ3a_ORF_19315 and AZ14_ORF_08205* in AZ_3a^T^ and AZ_14, respectively) encoding an aldolase and a kinase involved in the Entner-Doudoroff pathway, respectively (Table 2 and S1 Fig). Both genes were localized between *hylA* and *kduI* within the hyaluronate gene cluster described above. This was also true for *S*. *pneumoniae* TIGR4 (S1 Fig).

#### Iron uptake and metabolism

Three genes (AZ3a_ORF_18880, *pitA*; AZ3a_ORF_18870, *pitC* and AZ3a_ORF_18875, *pitD)* coding for structural components of a ferric iron ABC transporter and two genes *reg_RR* and *reg_SK* coding for a response regulator and a sensor kinase of a two-component system associated with the transporter, respectively, were acquired by both HV strains (Table 2 and S2 Fig). A similar operon was found in the three isogenic strains 1366, 2425 and 2426 (Table 2 and S2 Fig). Of note, a very similar operon was also identified in *S*. *mitis* strain NCTC12261 and *S*. *pneumoniae* TIGR 4 (S2 Fig).

#### Ascorbate utilization

The two HV strains harbored eight genes of a metabolic pathway transforming ascorbate in D-xylulose-5-phosphate (*AZ3a_ORF_21750 to AZ3a_ORF_21785* and *AZ14_ORF_08755 to AZ14_ORF_08790* in AZ_3a^T^ and AZ_14, respectively, Table 2, S1 Table and S3 Fig). Interestingly, this pathway was not found in the other strains described in this study (Table 2). This gene cluster inserts between a gene coding for sakacin-like protein and a transketolase (S3 Fig). Similar gene clusters were identified in several pathogens such as *S*. *pneumoniae*, *Streptococcus agalactiae*, *Streptococcus* suis, *Streptococcus uberis and Streptococcus pyogenes* (S3 Fig for *S*. *pneumoniae* TIGR4). Of note, no further genes were found between both genes in AZ_8 as well as *S*. *mitis* NTCC 12261 (S3 Fig).

#### Adhesion

**Fig 3:**
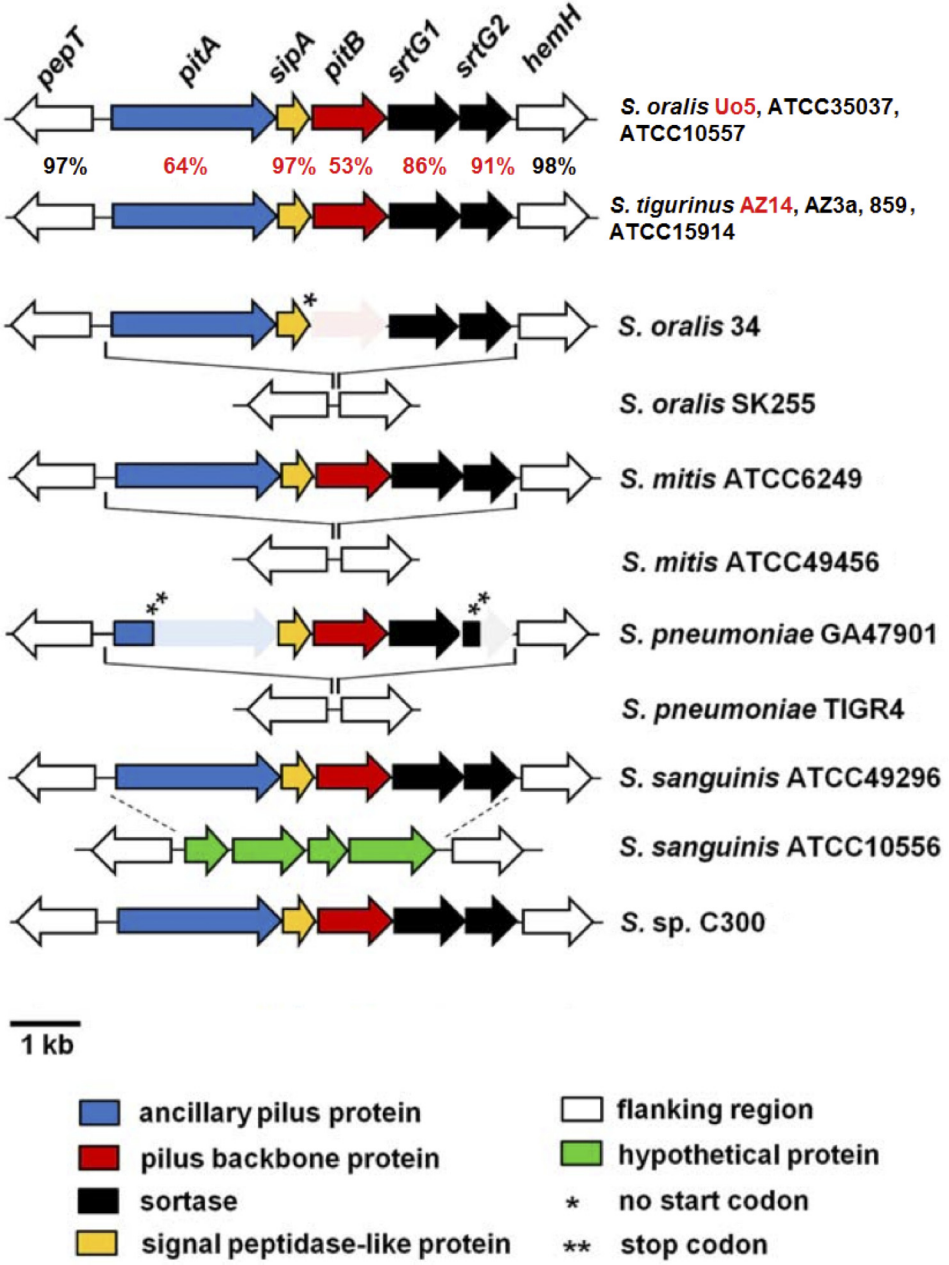
Alignment of PI-2 pilus islets, adapted from [22]. Genes are represented by colored arrows pointing in the direction of transcription. Gene names are indicated. % of identity are indicated for homologs found in *S*. *tigurinus* AZ_14 and *S*. *oralis* Uo5 (strains highlighted in red). % identity highlighted in red is those for the genes included in the PI-2 islet. Similarly as in all strains listed, the *S*. *tigurinus* PI-2 islets are inserted intergenically between *pepT* and *hemH*. Shaded areas indicate pseudogenes (one asterisk indicates a mutation in the start codon and two asterisks indicate the position of a stop-codon).

A region encompassing five ORFs (AZ3a_ORF_0110920 to AZ3a_ORF_11635 and AZ14_ORF_04295 to AZ14_ORF_04315 in AZ_3a^T^ and AZ_14, respectively) was found in both HV strains but not in the LV strain (Fig 3).

The first ORF with gene name *pitA* annotated as adhesin (587 Aa encoded by *AZ3a_ORF_0110920* and 844 Aa encoded by *AZ14_ORF_04295* in AZ_3a^T^ and AZ_14, respectively) was truncated at N-terminal part in AZ_3a^T^ removing a predicted VWA_2 domain, see below. The full length ORF could be retrieved from RAST annotation (862 Aa encoded by AZ3a_RAST_1017) (S4 Fig). AZ3a_RAST_1017 and AZ14_ORF_04295 share 86% identity and 100% coverage. They both harbor a von Willebrand factor type A domain (pfam13519, VWA_2) at the N-terminus and a sortase processing LPXTG-like motif (VPETG) at C-terminus (S4 Fig). Phobius predicted i) a signal peptide at position 1–35 for both proteins, ii) a non-cytoplasmic N-terminus (position 36–834 and 36–812 for AZ3a_RAST_1017 and AZ14_ORF_04295, respectively), iii) a transmembrane domain (position 835–857 and 813–840 for AZ3a_RAST_1017 and AZ14_ORF_04295, respectively) and iv) a cytoplasmic C-terminus (position 858–861 and 841–844 for AZ3a_RAST_1017 and AZ14_ORF_04295, respectively) (S4 Fig). Homologs showing 90%, 83% and 64% and 100% coverage with AZ14_ORF_04295 were present in *S*. *tigurinus* strains ATCC15914, *S*. *tigurinus* 859 and *S*. *oralis* Uo5, respectively (Table 2 and Fig 3). Moreover, homologs showing 87% identity and 76% coverage were annotated as fibronectin-binding proteins in several *S*. *pneumoniae* strains (not shown). Of note, despite *pitA* is a shared gene name for both the present adhesin and AZ3a_ORF_18880 of the gene cluster involved in iron uptake and metabolism (see above), both proteins are indeed distinct with different functions.

The second ORF, annotated as S26 family signal peptidase, was encoded by *AZ3a_ORF_11620* and by *AZ14_ORF_04300* in AZ_3a^T^ and AZ_14, respectively. Both homologs shared 99% identity and 100% coverage. Further homologs harboring 99% identity and 100% coverage were also found in many strains of *S*. *pneumoniae* (not shown).

The third ORF, annotated as a phosphate-transport permease PitB, was encoded by *AZ3a_ORF_11625* and by *AZ14_ORF_04305* in AZ_3a^T^ and AZ_14, respectively. Gene product homologs harbored 59% identity over 100% coverage (S5 Fig) and 53% identity over 100% coverage with a PitB protein of *S*. *oralis* Uo5 (Fig 3). As for the adhesin (first ORFs described above), Phobius predicted for both PitB-like homologs i) a signal peptide at position 1–40, ii) a non-cytoplasmic N-terminal (position 41–411 and 41–390 for AZ3a_ORF_11620 and AZ14_ORF_04305, respectively), iii) a transmembrane domain (position 412–429 and 391–408 for AZ3a_ORF_11620 and AZ14_ORF_04305, respectively) and iv) a cytoplasmic C-terminus (position 430–435 and 409–414 for AZ3a_ORF_11620 and AZ14_ORF_04305, respectively) (S5 Fig). Moreover both proteins harbored an FcTA conserved protein domain making them new members of the pfam 12892. Interestingly, other members of this family include fibronectin- and collagen-binding proteins.

Fourth ORF encoded by *AZ3a_ORF_11630* and *AZ14_ORF_04310* was annotated in NCBI as sortase SrtG1 and SrtB family sortase in AZ_3a^T^ and AZ_14, respectively. Both proteins harbored 99% identity and 100% coverage. Fifth ORF encoded by *AZ3a_ORF_11635* and *AZ14_ORF_04315* was annotated as SrtG1, sortase_B family protein and sortase in AZ_3a^T^ and AZ_14, respectively. Both proteins harbored 95% identity and 100% coverage. Of note, similar gene clusters were identified in *S*. *tigurinus* ATCC15914, *S*. *tigurinus* 895 and *S*. *oralis* Uo5 (Table 2).

### Genes specifically lost in the AZ8 LV strain

A total of 12 genes were predicted to be lost by the LV strain AZ_8. Besides isolated genes encoding for different enzymes (a methionine-R- sulfoxide reductase, an haloacid dehalogenase and a NUDIX hydrolase) and one sodium-dependent transporter (data not shown), six genes involved in tryptophan synthesis from chorismate (described below) were lost in AZ_8.

#### Chorismate to tryptophan pathway—indole production

Count predicted the loss by the LV strain AZ_8 of nearly all the genes—except *trpEB* and a truncated version of *trpB*—of a cluster encoding for enzymes involved in tryptophan synthesis from chorismate (Table 2 and S6 Fig). This gene cluster, that is maintained in both HV and other strains studied in this work, encompass *AZ3a_ORF_13845 to AZ3a_ORF_13875* and *AZ14_ORF_07355 to AZ14_ORF_07385* in AZ_3a^T^ and AZ_14, respectively (Table 2). A similar cluster was also identified in *Streptococcus gordonii* strain Challis substr. CH1 (S6 Fig).

## Discussion

*S*. *tigurinus* is a recently described streptococcal species responsible for severe invasive infections, including infective endocarditis, spondylodiscitis and meningitis [3–8]. As the identification of *S*. *tigurinus* by conventional phenotypic methods is limited, its contribution as a human pathogen is probably underestimated [3, 9]. Our group showed that infectivity was heterogeneous between *S*. *tigurinus* strains in an experimental model of infective endocarditis [10]. While some isolates yielded to severe infections (highly virulent, HV phenotype), other isolates were clearly less virulent (low virulent, LV phenotype). HV *S*. *tigurinus* strains clustered together and showed higher proteome similarity than LV or other strains. This finding suggests a more close relationship between these two HV strains, as shown from the phylogenetic analysis tree (Figs 1 and 2). This finding supports the hypothesis of some common genetic features between HV strains that are responsible of the exhibited high infectivity phenotype. Focusing on genes acquired by the two most virulent *S*. *tigurinus* strains AZ_3a^T^ and AZ_14, our comparative genomics analysis allowed us to identify genetic determinants potentially involved in enhanced infectivity in the model of experimental infective endocarditis in rat [10]. Of note, since our work focused on *S*. *tigurinus* intraspecies differences only, we limited the inclusion of *S*. *oralis* strains to the strain Uo5 that was identified as the closest strain to *S*. *tigurinus* species and served therefore as outgroup. In other words, adding other *S*. *oralis* strains to reflect the high heterogeneity of this species [23, 24] would not have modify the list of identified genetic differences between the *S*. *tigurinus* HV and LV strains.

### Hyaluronate utilization

A major finding was the presence of a complete operon coding for proteins involved in degradation and assimilation of a major constituent of the extracellular matrix throughout the human body, namely hyaluronate. Hyaluronate lyases which are the hyaluronate degrading enzymes [25] have often been considered as virulence factors in various bacterial pathogens [26, 27]. Besides, we identified in all strains a complete Entner-Doudoroff pathway that is involved in the metabolism of carbohydrates [28] and many transporters for host-tissue degradation products to be imported within the bacterial sacculus. Interestingly, Entner-Doudoroff pathway was shown to contribute to pathogenicity in *Vibrio cholerae* [29] and of highest importance regarding the present study, concomitant up regulation of this pathway with hyaluronate lyases was recently reported in invasive diseases induced by *Streptococcus dysgalactiae* [30]. It is therefore highly suspected that similar tissue-destroying enzymes and enzymes involved in carbohydrates metabolism and transport acting in concert significantly contribute to the high infectivity of HV *S*. *tigurinus* strains.

### Chorismate to tryptophan pathway—indole production

Presence of *trpAa* suggested that HV strains have the capacity to produce anthranilate from chorismate through the two-component enzymatic system TrpAa/TrpAb. In turns, TrpB, TrpC and TrpD—also absent from the LV strain—convert anthranilate into (3-Indoyl)-glycerol phosphate which is a precursor of both indole and tryptophan through two different reactions catalyzed by the same enzymatic complex formed by TrpEa and TrpEb [31]. Therefore, besides the fact that tryptophan might be an non-essential amino acid for HV strains, we believe that the maintained capacity to produce indole independently from the availability of exogenous tryptophan is highly relevant regarding regulation of pathogenicity of HV strains. Indeed, indole—which is mainly produced from L-tryptophan degradation by tryptophanases—is considered as an important intercellular signaling molecule sometime assimilated to a quorum sensing (QS) signal [32]. It has been shown to modulate biofilm production and expression of several virulence and antibiotic resistance genes and plays therefore a crucial role in the pathogenesis of several human bacterial pathogens [33]. Accordingly, such non-essential capacity for a strain of low virulence was lost in AZ_8.

### Ascorbate utilization

Our analysis identified an ascorbate metabolism pathway in both HV strains. To the best of our knowledge such pathway has not previously been linked to enhanced bacterial virulence. However, we believe that it could provide *S*. *tigurinus* with a relevant alternative carbon source possibly enhancing survival in blood in which ascorbic acid is available as a powerful antioxidant [34]. Indeed, D-Xylulose-5P—the end product of the ascorbate metabolism pathway—is the substrate of Xylulose-5P phosphoketolase to produce D-Glyceraldehyde-3P that in turn enters glycolysis. Regarding potential implication of this pathway in the HV phenotype, it is likely that increased survival in blood would correlate with increased chances for the bacteria to establish endocarditis.

### Iron uptake

In the same manner and as described in *S*. *aureus* for *isd* genes [35], we hypothesize that, besides the iron uptake ABC transporter coded by *piuABCD* present in all strains, the presence of *pitACD* encoding for an additional ferric ABC transporter allows HV strains to efficiently capture free iron in blood. It is known that concentration of free iron in blood usually accessible for bacteria is below the concentration required for bacterial survival [35]. However, despite the fact that under normal condition most transition metal ions are not free but attached to carrier proteins [36–38], under pathological conditions release of metal ions from their binding protein ferritin can occur [39]. Therefore, similarly to the capacity to metabolize ascorbate, improved capacity to capture free irons could lead to increased survival of HV strains in blood and subsequent capacity to induce infective endocarditis.

### Adhesion

We believe that the gene clusters spanning *AZ3a_ORF_0110920* to *AZ3a_ORF_11635* and *AZ14_ORF_04295* to *AZ14_ORF_04305* in AZ_3a^T^ and AZ_14, respectively correspond to a PI-2-like pilus islets previously described in several streptococcal species among which *S*. *pneumoniae*, *S*. *mitis* and *S*. *sanguinis* [22]. Indeed, the genetic organization of the islets identified in *S*. *tigurinus* AZ_3a^T^, AZ_14, ATCC15914 and 859 is very similar to the previously described PI-2 pilus islets. The gene product encoded by the first ORF harbor a VWA_2 domain at the N-terminus and a sortase processing LPXTG-like motif (VPETG) at C-terminus. This protein could therefore well represent a PitA homolog since PitA has been shown to harbor such domains in several *S*. *pneumoniae* strains [40]. Moreover, VWA_2 domains have been identified in Gram-positive pilus proteins having adhesive functions [41–44] and LPXTG proteins are involved in various functions, including host colonization in which they play crucial roles in bacterial adhesion to host tissues, and are often termed adhesins [45]. Therefore it is very likely that *AZ3a_RAST_1017* and *AZ14_ORF_04295* encode for a PitA homolog with adhesive function. Similarly the third ORF (i.e. *AZ3a_ORF_11625* and *AZ14_ORF_0305* in AZ_3a^T^ and AZ_14, respectively) encode for proteins harboring a non-canonical LPXTG-like motif (VTPTG). Regarding genomic localization and since VTPTG motifs have been reported in pilus backbone proteins PitB from several other streptococci [22, 44], it is highly likely that both proteins represent PitB homologs. The second ORFs potentially encode for S26 family signal peptidase SipA homologs and accordingly harbor peptidase_26 domains (pfam 10512). Members of this protein family are essential membrane-bound serine proteases that function to cleave the amino-terminal signal peptide extension from proteins that are translocated across biological membranes. SipA has been shown to be required for biosynthesis of pilus in *S*. *pyogenes* [46] and very interestingly, expression of a *S*. *suis* SipA homolog was found to be highly upregulated when the bacterium interacted with brain microvascular endothelial cells, suggesting a role in the infection process of this pathogen [47]. Finally, as found in other PI-2 pilus islets, two sortases of the B class, SrtG1 and SrtG2, were present on *S*. *tigurinus* PI-2 pilus islets. Sortases are classified within families of classes A–F [48]. These enzymes are involved in cell adhesion, iron acquisition, and spore formation [49]. Sortase A recognize LPXTG sorting motifs to anchor surface proteins to the cell wall envelope [50] and sortase B recognize variants of this canonical LPXTG motif such as NPQTN or NPKTG depending on the organism [51, 52]. While the role of the dispensable SrtG2 remains unclear, SrtG1 was found to catalyze the covalent association of PitB monomers [44, 53]. Taken together, these results indicating the presence of PI-2 pilus islets in HV but not in LV strains appears very relevant regarding the phenotype of highly infectivity of the HV strains. Indeed, such PI-2 pili have been shown to mediate adhesion of *S*. *pneumoniae* to eukaryotic cells [44] and were proposed as candidates for contributing to adhesive interactions in *S*. *mitis* [22]. To the best of our knowledge, this is first report of PI-2-like pilus islets in *S*. *tigurinus*.

## Conclusions

Using comparative genomics approaches, we were able to identify several genetic features acquired only by the two *S*. *tigurinus* HV strains AZ-3a^T^ and AZ_14 but not present in the *S*. *tigurinus* LV strain AZ_8. We believe that the identified features could largely explain the phenotype of high virulence previously observed for both HV strains in an experimental model of infective endocarditis in rats. Indeed, these features include determinants that could be involved at different stages of the disease. First of all, determinants involved in iron uptake and ascorbate metabolism could directly promote survival of *S*. *tigurinus* in blood. Second, specific adhesins encoded on new PI-2-like islets could mediate initial attachment of bacterial pathogen to the damaged cardiac tissue and/or vegetation that formed on site. Third, newly acquired tissue-destroying enzymes (hyaluronate operon), enzymes involved in carbohydrates metabolism (Entner-Doudoroff pathway) and associated transporters could act in concert to increase tissue invasion. In addition, some of these steps could well be coordinated within the pathogenic population through a specific indole based QS. Obviously, these hypotheses need experimental validations, but our list of genes potentially responsible for the increased infectivity of HV strains represents relevant genetic determinants to explore further. For instance, deletion mutants could be evaluated in our model of infective endocarditis. Moreover, if correlation between our findings and experimental results is demonstrated, new PCR assays targeting HV specific genes aiming at predicting the infectivity potential of any *S*. *tigurinus* isolate could well be developed.

## Supporting Information

S1 Fig Gene cluster involved in hyaluronate metabolism and Entner-Doudoroff pathway. This cluster is found in the highly virulent (HV) strains AZ_3a^T^ and AZ_14 but absent in the low virulent (LV) strain AZ_8. A similar cluster is found on *S*. *pneumoniae* TIGR4. Genes are represented by colored arrows pointing in the direction of transcription. Gene names are indicated.
(TIFF)

S2 Fig *pitACD* gene cluster involved in iron uptake. This cluster is found in highly virulent (HV) strains AZ_3a^T^ and AZ_14 but absent in the low virulent (LV) strain AZ_8. A similar gene cluster was found in *S*. *mitis* NCTC 12261 and *S*. *pneumoniae* TIGR 4. Genes are represented by colored arrows pointing in the direction of transcription. Gene names are indicated. The cluster is localized between a gene coding for a hypothetical protein (red arrow) and a tRNA leucine synthase.
(TIFF)

S3 Fig Gene cluster involved in ascorbate metabolism. This cluster is found in highly virulent (HV) strains AZ_3a^T^ and AZ_14 but absent in all other strains included in the present study. A similar gene cluster was found in *S*. *pneumoniae* TIGR 4. Genes are represented by colored arrows pointing in the direction of transcription. Gene names are indicated. The cluster is localized between a gene coding for sakacin (red arrow) and a transketolase (purple arrow).
(TIFF)

S4 Fig Clustal Omega alignment of the PitA-like protein homologs. The LPXTG-like sortase processing motifs (VPETG) are highlighted in bold and underscored. The 3 conserved residues (DTD) found in the MIDAS feature of the identified N-terminal vWA_2 domain (pfam 13529) are highlighted in bold. Transmembrane domains identified by Phobius are highlighted in bold and in italic at C-terminus.
(TIFF)

S5 Fig Clustal Omega alignment of the PitB-like protein homologs. The non-canonical LPXTG-like sortase processing motifs (VTPTG) are highlighted in bold and underscored. Transmembrane domains identified by Phobius are highlighted in bold and in italic at C-terminus.
(TIFF)

S6 Fig Gene cluster involved in chorismate to tryptophan metabolism. This cluster is found in highly virulent (HV) strains AZ_3a^T^ and AZ_14 but absent in the low virulent (LV) strain AZ_8. A similar cluster is found in *S*. *gordonii* strain Challis substr. CH1. Genes are represented by colored arrows pointing in the direction of transcription. Gene names are indicated. The cluster is localized between *msba* coding for a lipid A permease and *comC* coding for a processing protease involved in competence. In AZ_8 only a single copy of *trpEb* and a truncated version of *trpB* are present.
(TIFF)

S1 Table Full list of the 188 genes present in the highly virulent (HV) strains AZ_3a^T^ and AZ_14 but absent in the low virulent (LV) strain AZ_8. This list has been obtained using the Family History by Dollo Parsimony tool of Count Software. Genomic regions of particular interest further investigated in the present study are highlighted in light grey.
(DOCX)
