# Supplementary figures and images for: Comparative Genomics Analysis of Streptococcus tigurinus Strains Identifies Genetic Elements Specifically and Uniquely Present in Highly Virulent Strains
Source: PLoS One. 2016 Aug 9;11(8):e0160554. doi: 10.1371/journal.pone.0160554 (PMC4978470; doi:10.1371/journal.pone.0160554)

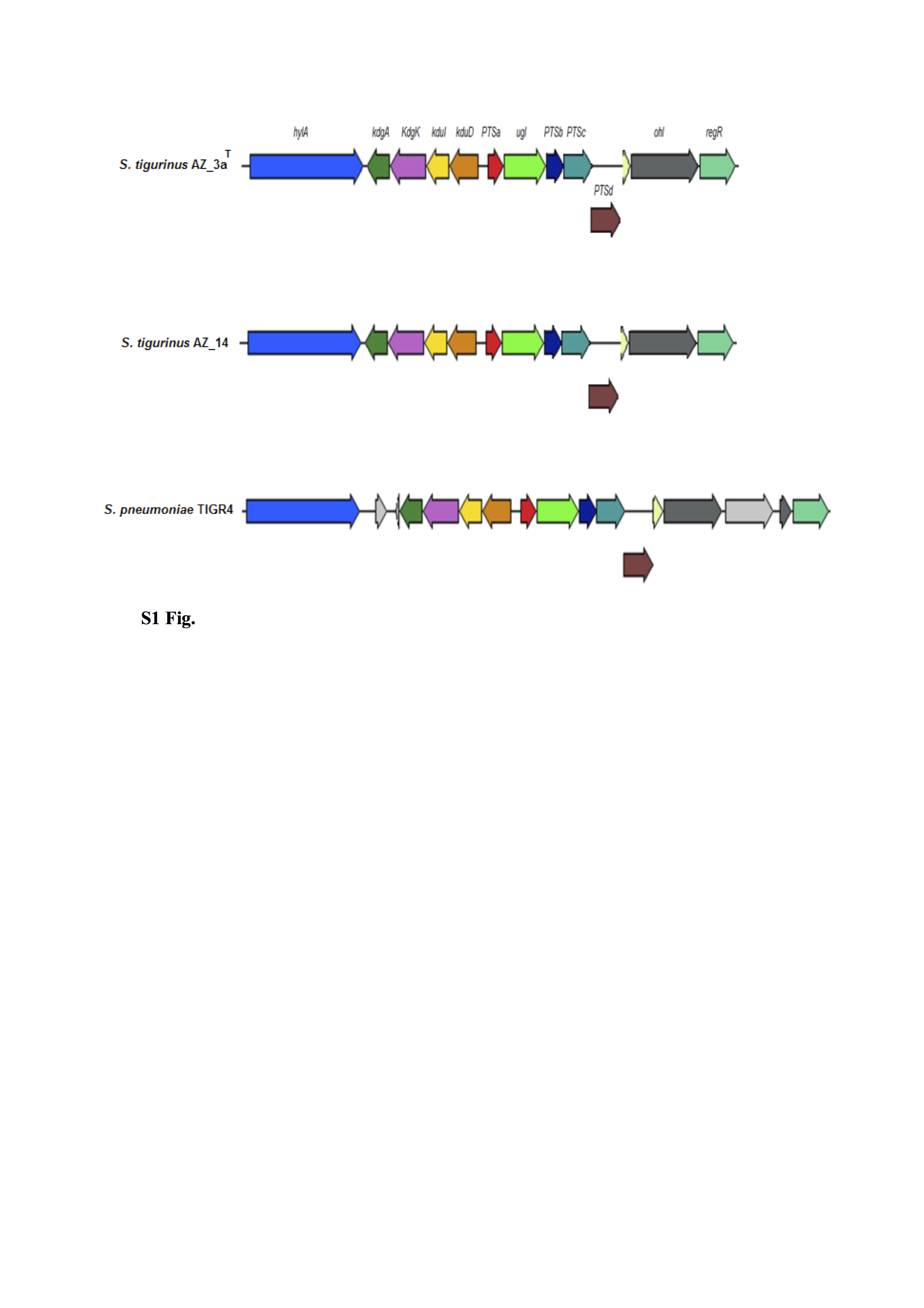

Supplement: S1 Fig — This cluster is found in the highly virulent (HV) strains AZ_3aT and AZ_14 but absent in the low virulent (LV) strain AZ_8. A similar cluster is found on S. pneumoniae TIGR4. Genes are represented by colored arrows pointing in the direction of transcription. Gene names are indicated. (TIFF) [file pone.0160554.s001.tiff]

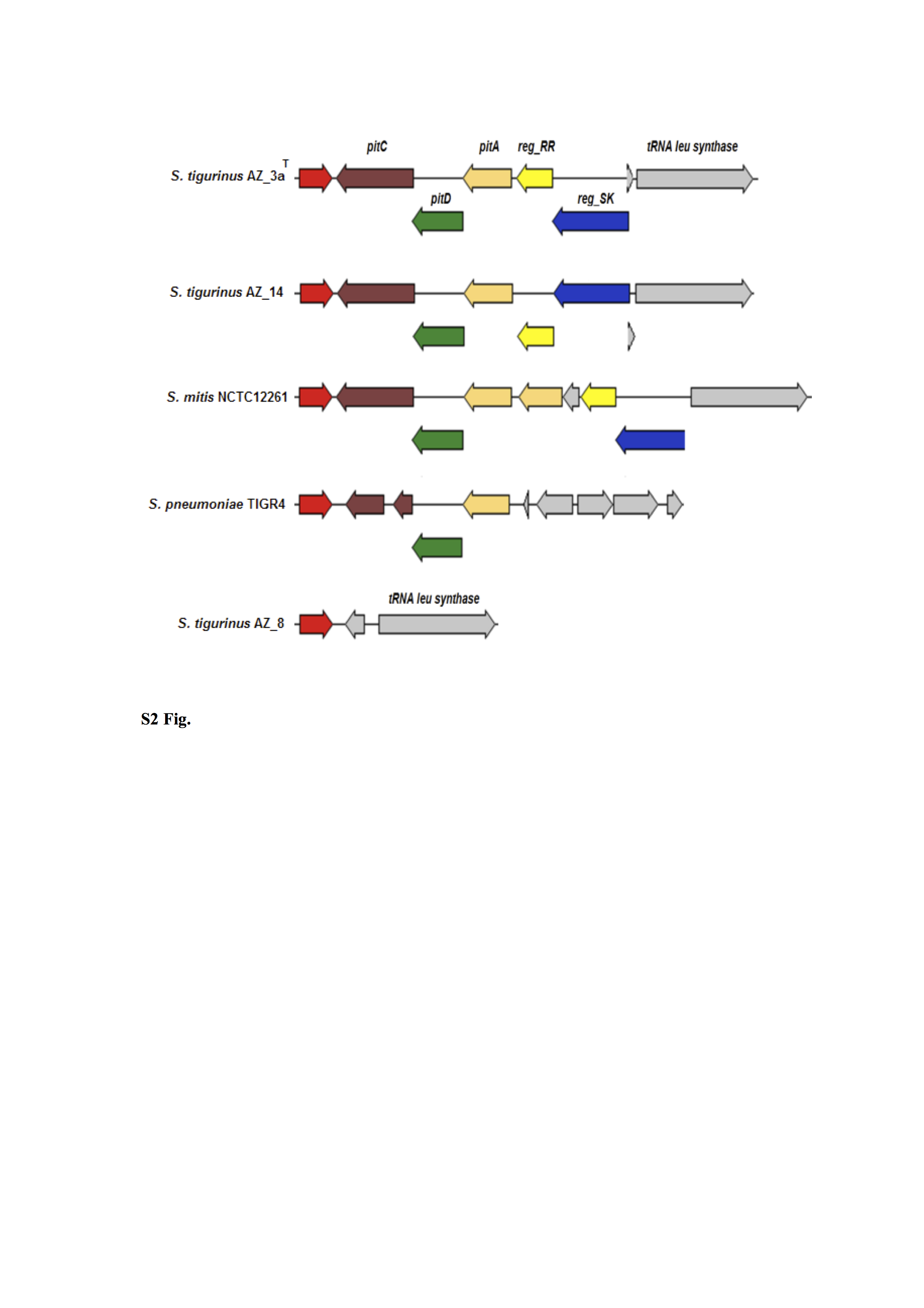

Supplement: S2 Fig — This cluster is found in highly virulent (HV) strains AZ_3aT and AZ_14 but absent in the low virulent (LV) strain AZ_8. A similar gene cluster was found in S. mitis NCTC 12261 and S. pneumoniae TIGR 4. Genes are represented by colored arrows pointing in the direction of transcription. Gene names are indicated. The cluster is localized between a gene coding for a hypothetical protein (red arrow) and a tRNA leucine synthase. (TIFF) [file pone.0160554.s002.tiff]

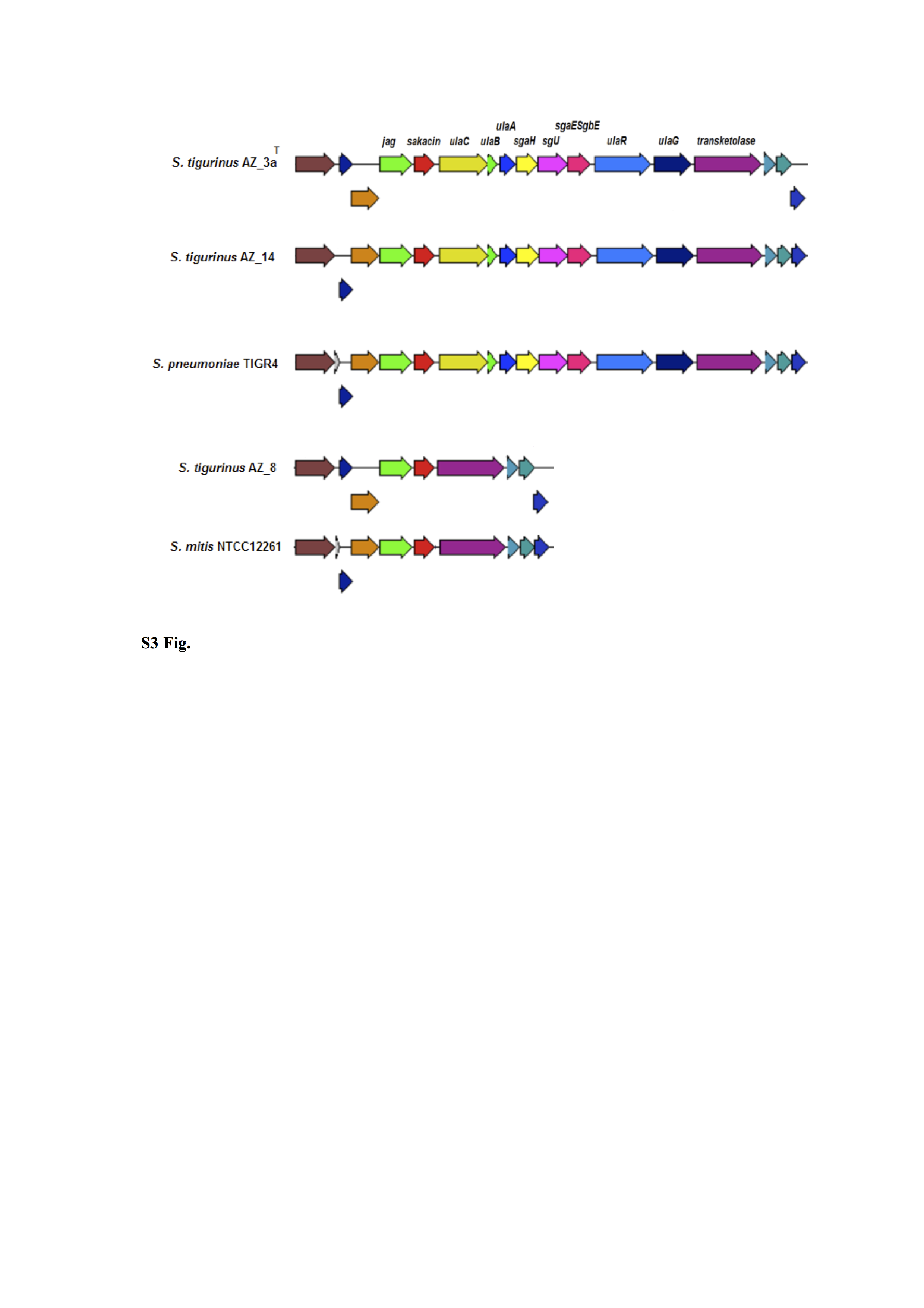

Supplement: S3 Fig — This cluster is found in highly virulent (HV) strains AZ_3aT and AZ_14 but absent in all other strains included in the present study. A similar gene cluster was found in S. pneumoniae TIGR 4. Genes are represented by colored arrows pointing in the direction of transcription. Gene names are indicated. The cluster is localized between a gene coding for sakacin (red arrow) and a transketolase (purple arrow). (TIFF) [file pone.0160554.s003.tiff]

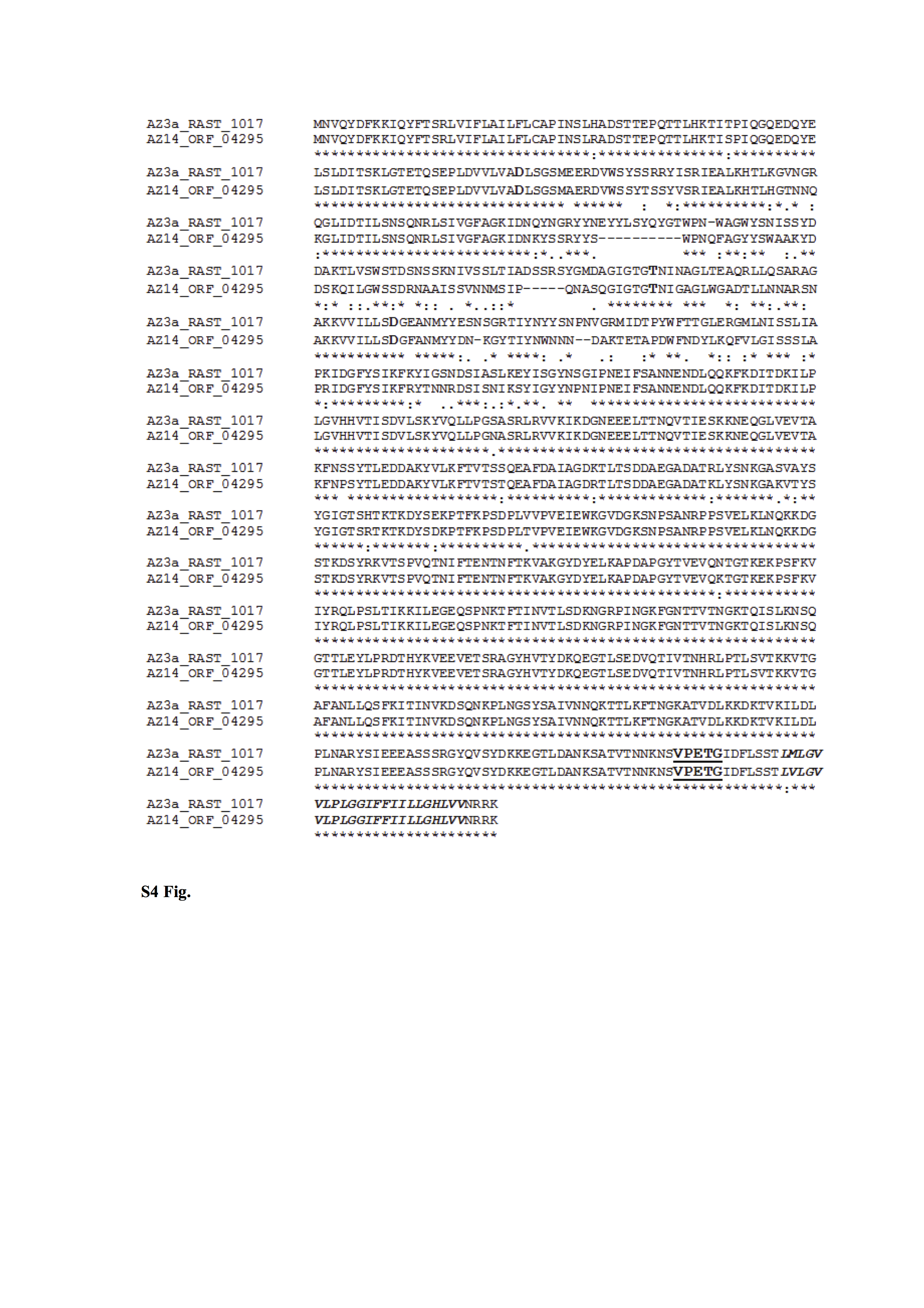

Supplement: S4 Fig — The LPXTG-like sortase processing motifs (VPETG) are highlighted in bold and underscored. The 3 conserved residues (DTD) found in the MIDAS feature of the identified N-terminal vWA_2 domain (pfam 13529) are highlighted in bold. Transmembrane domains identified by Phobius are highlighted in bold and in italic at C-terminus. (TIFF) [file pone.0160554.s004.tiff]

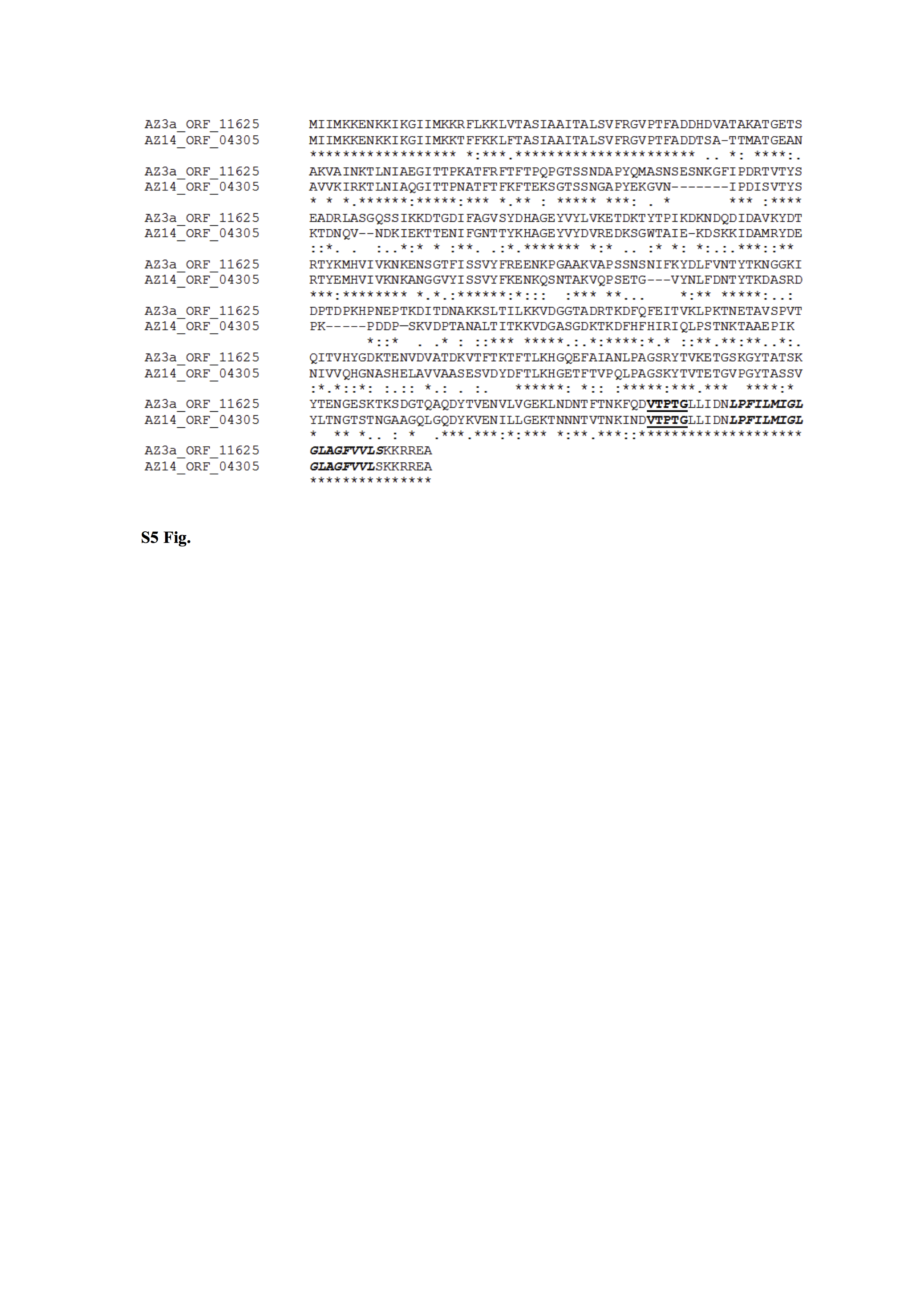

Supplement: S5 Fig — The non-canonical LPXTG-like sortase processing motifs (VTPTG) are highlighted in bold and underscored. Transmembrane domains identified by Phobius are highlighted in bold and in italic at C-terminus. (TIFF) [file pone.0160554.s005.tiff]

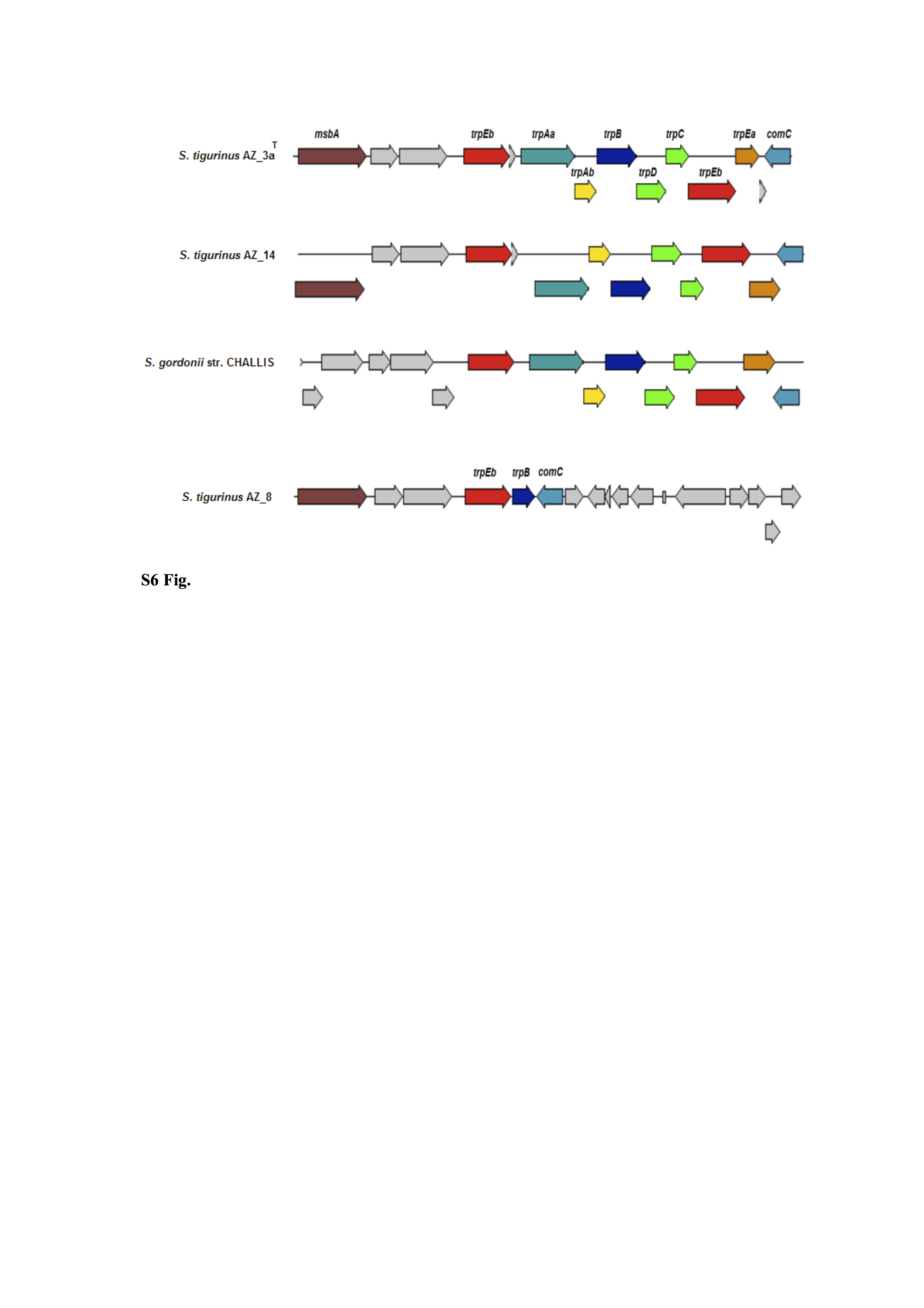

Supplement: S6 Fig — This cluster is found in highly virulent (HV) strains AZ_3aT and AZ_14 but absent in the low virulent (LV) strain AZ_8. A similar cluster is found in S. gordonii strain Challis substr. CH1. Genes are represented by colored arrows pointing in the direction of transcription. Gene names are indicated. The cluster is localized between msba coding for a lipid A permease and comC coding for a processing protease involved in competence. In AZ_8 only a single copy of trpEb and a truncated version of trpB are present. (TIFF) [file pone.0160554.s006.tiff]
