# Supplementary material for: Comparative Genomics Analysis of Streptococcus tigurinus Strains Identifies Genetic Elements Specifically and Uniquely Present in Highly Virulent Strains
Source: PLoS One. 2016 Aug 9;11(8):e0160554. doi: 10.1371/journal.pone.0160554 (PMC4978470; doi:10.1371/journal.pone.0160554)
Supplement: S1 Table — This list has been obtained using the Family History by Dollo Parsimony tool of Count Software. Genomic regions of particular interest further investigated in the present study are highlighted in light grey. (DOCX) [file pone.0160554.s007.docx]

**S1 Table. Full list of the 188 genes present in the highly virulent (HV) strains AZ_3a^T^ and AZ_14 but absent in the low virulent (LV) strain AZ_8.** This list has been obtained using the Family History by Dollo Parsimony tool of Count Software. Genomic regions of particular interest further investigated in the present study are highlighted in light grey.

| **ORF N° in AZ_3a^T^** | **ORF N° in AZ_14** | **Gene**  **name** | **Annotated function in NCBI for AZ_3a^T^** |
| --- | --- | --- | --- |
| AZ3a_ORF_11610 | AZ14_ORF_04280 |  | histidine triad (HIT) protein |
| AZ3a_ORF_0110920 | AZ14_ORF_04295 | *pitA* | adhesin |
| AZ3a_ORF_11620 | AZ14_ORF_04300 | *sipA* | S26 family signal peptidase |
| AZ3a_ORF_11625 | AZ14_ORF_04305 | *pitB* | phosphate-transport permease PitB |
| AZ3a_ORF_11630 | AZ14_ORF_04310 | *srtG1* | sortase SrtG1 |
| AZ3a_ORF_11635 | AZ14_ORF_04315 | *srtG2* | sortase |
| AZ3a_ORF_11735 | AZ14_ORF_04420 |  | hypothetical protein |
| AZ3a_ORF_11740 | AZ14_ORF_04425 |  | replication initiation protein |
| AZ3a_ORF_11745 | AZ14_ORF_04430 |  | nucleoside triphosphate hydrolase |
| AZ3a_ORF_11750 | AZ14_ORF_04435 |  | conjugal transfer protein |
| AZ3a_ORF_11760 | AZ14_ORF_08110 |  | recombinase |
| AZ3a_ORF_11765 | AZ14_ORF_08105 |  | alpha/beta hydrolase |
| AZ3a_ORF_11775 | AZ14_ORF_08095 |  | conjugal transfer protein TraA |
| AZ3a_ORF_11780 | AZ14_ORF_08090 |  | conjugal transfer protein |
| AZ3a_ORF_11790 | AZ14_ORF_08080 |  | transposase |
| AZ3a_ORF_11800 | AZ14_ORF_04445 |  | recombinase TnpX |
| AZ3a_ORF_11805 | AZ14_ORF_04450 |  | TetR family transcriptional regulator |
| AZ3a_ORF_11810 | AZ14_ORF_04455 |  | cytoplasmic protein |
| AZ3a_ORF_11830 | AZ14_ORF_04475 |  | cobalt transporter |
| AZ3a_ORF_11835 | AZ14_ORF_04480 |  | hypothetical protein |
| AZ3a_ORF_11840 | AZ14_ORF_04485 |  | RNA polymerase subunit sigma-70 |
| AZ3a_ORF_11845 | AZ14_ORF_04490 |  | hypothetical protein |
| AZ3a_ORF_11860 | AZ14_ORF_04505 |  | hypothetical protein |
| AZ3a_ORF_11865 | AZ14_ORF_04510 |  | DNA-binding protein |
| AZ3a_ORF_11870 | AZ14_ORF_04515 |  | transposase |
| AZ3a_ORF_11875 | AZ14_ORF_04520 |  | conjugal transfer protein TraG |
| AZ3a_ORF_11880 | AZ14_ORF_04525 |  | single-stranded DNA-binding protein |
| AZ3a_ORF_11930 | AZ14_ORF_04495 |  | MobA/MobL protein |
| AZ3a_ORF_11960 | AZ14_ORF_04535 |  | hypothetical protein |
| AZ3a_ORF_11965 | AZ14_ORF_04540 |  | conjugal transfer protein |
| AZ3a_ORF_11970 | AZ14_ORF_04545 |  | conjugal transfer protein |
| AZ3a_ORF_11980 | AZ14_ORF_04585 |  | group II intron reverse transcriptase/maturase |
| AZ3a_ORF_11995 | AZ14_ORF_04560 |  | conjugal transfer protein |
| AZ3a_ORF_12005 | AZ14_ORF_04570 |  | DNA topoisomerase III |
| AZ3a_ORF_12015 | AZ14_ORF_04580 |  | conjugal transfer protein |
| AZ3a_ORF_12020 | AZ14_ORF_04595 |  | DNA-binding protein |
| AZ3a_ORF_12050 | AZ14_ORF_04605 |  | conjugal transfer protein |
| AZ3a_ORF_12070 | AZ14_ORF_04675 |  | RNA polymerase subunit sigma |
| AZ3a_ORF_12095 | AZ14_ORF_04750 |  | phosphomethylpyrimidine kinase |
| AZ3a_ORF_12100 | AZ14_ORF_04760 |  | multidrug transporter |
| AZ3a_ORF_12110 | AZ14_ORF_04765 |  | hypothetical protein |
| AZ3a_ORF_12145 | AZ14_ORF_04785 |  | hypothetical protein |
| AZ3a_ORF_12190 | AZ14_ORF_04830 |  | MarR family transcriptional regulator |
| AZ3a_ORF_12195 | AZ14_ORF_04835 |  | hypothetical protein |
| AZ3a_ORF_12240 | AZ14_ORF_04880 |  | hypothetical protein |
| AZ3a_ORF_12300 | AZ14_ORF_04940 |  | hypothetical protein |
| AZ3a_ORF_12305 | AZ14_ORF_04945 |  | hypothetical protein |
| AZ3a_ORF_12310 | AZ14_ORF_04950 |  | hypothetical protein |
| AZ3a_ORF_12320 | AZ14_ORF_04960 |  | peptidase |
| AZ3a_ORF_12325 | AZ14_ORF_04965 |  | hypothetical protein |
| AZ3a_ORF_12330 | AZ14_ORF_04970 |  | exopolysaccharide biosynthesis protein |
| AZ3a_ORF_12335 | AZ14_ORF_04975 |  | sugar translocase |
| AZ3a_ORF_12425 | AZ14_ORF_05065 |  | hypothetical protein |
| AZ3a_ORF_12525 | AZ14_ORF_05165 |  | acetyltransferase |
| AZ3a_ORF_12680 | AZ14_ORF_05335 |  | endonuclease |
| AZ3a_ORF_12930 | AZ14_ORF_05590 |  | thiol-activated cytolysin |
| AZ3a_ORF_13435 | AZ14_ORF_07140 |  | XRE family transcriptional regulator |
| AZ3a_ORF_13445 | AZ14_ORF_07150 |  | choline-binding protein A |
| AZ3a_ORF_13805 | AZ14_ORF_07315 |  | PTS mannose transporter subunit IIA |
| AZ3a_ORF_13810 | AZ14_ORF_07320 |  | PTS sugar transporter subunit IIB |
| AZ3a_ORF_13815 | AZ14_ORF_07325 |  | PEP phosphonomutase |
| AZ3a_ORF_13820 | AZ14_ORF_07330 |  | PTS cellobiose transporter subunit IIC |
| AZ3a_ORF_13825 | AZ14_ORF_07335 |  | MarR family transcriptional regulator |
| AZ3a_ORF_13845 | AZ14_ORF_07355 | *trpEb* | tryptophan synthase subunit beta |
| AZ3a_ORF_13850 | AZ14_ORF_07360 | *trpC* | N-(5'-phosphoribosyl)anthranilate isomerase |
| AZ3a_ORF_13855 | AZ14_ORF_07365 | *trpD* | indole-3-glycerol phosphate synthase |
| AZ3a_ORF_13860 | AZ14_ORF_07370 | *trpB* | anthranilate phosphoribosyltransferase |
| AZ3a_ORF_13870 | AZ14_ORF_07380 | *trpAa* | anthranilate synthase |
| AZ3a_ORF_13875 | AZ14_ORF_07385 |  | hypothetical protein |
| AZ3a_ORF_13940 | AZ14_ORF_07465 |  | carbohydrate phosphatase |
| AZ3a_ORF_13950 | AZ14_ORF_07475 |  | LacI family transcriptional regulator |
| AZ3a_ORF_13955 | AZ14_ORF_07480 |  | Zn-dependent alcohol dehydrogenase |
| AZ3a_ORF_13960 | AZ14_ORF_07485 |  | MerR family transcriptional regulator |
| AZ3a_ORF_14185 | AZ14_ORF_03325 |  | CAAX protease |
| AZ3a_ORF_14475 | AZ14_ORF_03025 |  | small molecule-binding protein |
| AZ3a_ORF_14480 | AZ14_ORF_03020 |  | DNA-binding transcriptional regulator |
| AZ3a_ORF_14535 | AZ14_ORF_02965 |  | permease |
| AZ3a_ORF_14540 | AZ14_ORF_02960 |  | hypothetical protein |
| AZ3a_ORF_14545 | AZ14_ORF_02955 |  | choline kinase |
| AZ3a_ORF_14620 | AZ14_ORF_02880 |  | sodium-dependent transporter |
| AZ3a_ORF_15035 | AZ14_ORF_02470 |  | ABC transporter |
| AZ3a_ORF_15040 | AZ14_ORF_02465 |  | ABC transporter ATP-binding protein |
| AZ3a_ORF_15045 | AZ14_ORF_02460 |  | AbrB family transcriptional regulator |
| AZ3a_ORF_15050 | AZ14_ORF_02455 |  | ROK family transcriptional regulator |
| AZ3a_ORF_15480 | AZ14_ORF_02045 |  | peptidase |
| AZ3a_ORF_15580 | AZ14_ORF_01955 |  | GntR family transcriptional regulator |
| AZ3a_ORF_15585 | AZ14_ORF_01950 |  | hypothetical protein |
| AZ3a_ORF_15590 | AZ14_ORF_01945 |  | hypothetical protein |
| AZ3a_ORF_15595 | AZ14_ORF_01940 |  | ABC transporter |
| AZ3a_ORF_15600 | AZ14_ORF_01935 |  | TetR family transcriptional regulator |
| AZ3a_ORF_15785 | AZ14_ORF_00835 |  | hypothetical protein |
| AZ3a_ORF_15795 | AZ14_ORF_00825 |  | MutR family transcriptional regulator |
| AZ3a_ORF_16040 | AZ14_ORF_00575 |  | ABC transporter ATP-binding protein |
| AZ3a_ORF_16145 | AZ14_ORF_00485 |  | GTP-binding protein |
| AZ3a_ORF_16150 | AZ14_ORF_00480 |  | replication protein |
| AZ3a_ORF_16160 | AZ14_ORF_00465 |  | hypothetical protein |
| AZ3a_ORF_16170 | AZ14_ORF_00460 |  | hypothetical protein |
| AZ3a_ORF_16230 | AZ14_ORF_00300 |  | cell wall anchor protein |
| AZ3a_ORF_16410 | AZ14_ORF_00115 |  | bacteriocin leader domain-containing protein |
| AZ3a_ORF_16505 | AZ14_ORF_05895 |  | integrase |
| AZ3a_ORF_16510 | AZ14_ORF_05900 |  | hypothetical protein |
| AZ3a_ORF_16515 | AZ14_ORF_05905 |  | hypothetical protein |
| AZ3a_ORF_16525 | AZ14_ORF_05915 |  | hypothetical protein |
| AZ3a_ORF_16530 | AZ14_ORF_05920 |  | calcium-binding protein |
| AZ3a_ORF_16540 | AZ14_ORF_05930 |  | abortive phage infection protein |
| AZ3a_ORF_16545 | AZ14_ORF_05935 |  | abortive phage infection protein |
| AZ3a_ORF_16560 | AZ14_ORF_05940 |  | hypothetical protein |
| AZ3a_ORF_16570 | AZ14_ORF_05950 |  | transcriptional regulator |
| AZ3a_ORF_16585 | AZ14_ORF_05965 |  | hypothetical protein |
| AZ3a_ORF_16590 | AZ14_ORF_05970 |  | conjugal transfer protein TrbL |
| AZ3a_ORF_16595 | AZ14_ORF_05975 |  | hypothetical protein |
| AZ3a_ORF_16605 | AZ14_ORF_05985 |  | hypothetical protein |
| AZ3a_ORF_16610 | AZ14_ORF_05990 |  | abortive phage infection protein |
| AZ3a_ORF_16615 | AZ14_ORF_05995 |  | hypothetical protein |
| AZ3a_ORF_16620 | AZ14_ORF_06000 |  | arsenate reductase |
| AZ3a_ORF_16625 | AZ14_ORF_06005 |  | hypothetical protein |
| AZ3a_ORF_16630 | AZ14_ORF_06010 |  | DNA cytosine methyltransferase |
| AZ3a_ORF_16635 | AZ14_ORF_06015 |  | replication initiator protein |
| AZ3a_ORF_16665 | AZ14_ORF_06045 |  | peptide methionine sulfoxide reductase |
| AZ3a_ORF_16950 | AZ14_ORF_06335 |  | SAM-dependent methyltransferase |
| AZ3a_ORF_17050 | AZ14_ORF_06435 |  | AAA family ATPase |
| AZ3a_ORF_17220 | AZ14_ORF_06595 |  | serine protease |
| AZ3a_ORF_17505 | AZ14_ORF_01630 |  | hypothetical protein |
| AZ3a_ORF_18225 | AZ14_ORF_00415 |  | cell division protein FtsK |
| AZ3a_ORF_18230 | AZ14_ORF_00410 |  | Cro/Cl family transcriptional regulator |
| AZ3a_ORF_18295 | AZ14_ORF_00390 |  | ATP/GTP-binding protein |
| AZ3a_ORF_18560 | AZ14_ORF_00955 |  | XRE family transcriptional regulator |
| AZ3a_ORF_18870 | AZ14_ORF_01270 | *pitC* | iron ABC transporter permease |
| AZ3a_ORF_18875 | AZ14_ORF_01275 | *pitD* | peptide ABC transporter substrate-binding protein |
| AZ3a_ORF_18880 | AZ14_ORF_01280 | *pitA* | iron ABC transporter substrate-binding protein |
| AZ3a_ORF_18890 | AZ14_ORF_01290 | *reg_SK* | histidine kinase |
| AZ3a_ORF_22340 | AZ14_ORF_01285 | *reg_RR* | AraC family transcriptional regulator |
| AZ3a_ORF_18900 | AZ14_ORF_01300 |  | transcriptional regulator |
| AZ3a_ORF_18905 | AZ14_ORF_01305 |  | acyl-CoA thioesterase |
| AZ3a_ORF_19120 | AZ14_ORF_00500 |  | hypothetical protein |
| AZ3a_ORF_19250 | AZ14_ORF_01390 |  | 5,10-methylene tetrahydromethanopterin reductase |
| AZ3a_ORF_19305 | AZ14_ORF_08215 | *hylA* | hyaluronate lyase |
| AZ3a_ORF_19310 | AZ14_ORF_08210 | *kdgA* | keto-deoxy-phosphogluconate aldolase |
| AZ3a_ORF_19315 | AZ14_ORF_08205 | *kdgK* | 2-keto-3-deoxygluconate kinase |
| AZ3a_ORF_19320 | AZ14_ORF_08200 | *kduI* | hypothetical protein |
| AZ3a_ORF_19325 | AZ14_ORF_08195 | *kduD* | Gluconate 5-dehydrogenase |
| AZ3a_ORF_19330 | AZ14_ORF_08190 | *PTSa* | PTS N-acetylgalactosamine transporter subunit IIA |
| AZ3a_ORF_19335 | AZ14_ORF_08185 | *ugl* | glucuronyl hydrolase |
| AZ3a_ORF_19340 | AZ14_ORF_08180 | *PTSb* | PTS N-acetylgalactosamine transporter subunit IIB |
| AZ3a_ORF_19345 | AZ14_ORF_08175 | *PTSc* | PTS N-acetylgalactosamine transporter subunit IIC |
| AZ3a_ORF_19350 | AZ14_ORF_08170 | *PTSd* | PTS N-acetylgalactosamine transporter subunit IID |
| AZ3a_ORF_19355 | AZ14_ORF_08165 |  | preprotein translocase subunit YajC |
| AZ3a_ORF_19360 | AZ14_ORF_08160 | *ohl* | oligohyaluronate lyase |
| AZ3a_ORF_19365 | AZ14_ORF_08155 | *regR* | transcriptional regulator |
| AZ3a_ORF_19545 | AZ14_ORF_07925 |  | 4-methyl-5(B-hydroxyethyl)-thiazole monoP BP |
| AZ3a_ORF_19650 | AZ14_ORF_07825 |  | Hypothetical protein |
| AZ3a_ORF_19660 | AZ14_ORF_07815 |  | Flavin reductase |
| AZ3a_ORF_19865 | AZ14_ORF_09285 |  | transporter |
| AZ3a_ORF_19870 | AZ14_ORF_09290 |  | hypothetical protein |
| AZ3a_ORF_19975 | AZ14_ORF_09400 |  | hypothetical protein |
| AZ3a_ORF_19980 | AZ14_ORF_09405 |  | hypothetical protein |
| AZ3a_ORF_19990 | AZ14_ORF_09415 |  | 2-dehydropantoate 2-reductase |
| AZ3a_ORF_20460 | AZ14_ORF_03670 |  | Hypothetical protein |
| AZ3a_ORF_20465 | AZ14_ORF_03665 |  | NUDIX hydrolase |
| AZ3a_ORF_20650 | AZ14_ORF_08495 |  | Hypothetical protein |
| AZ3a_ORF_20750 | AZ14_ORF_08395 |  | DNA-directed RNA polymerase subunit beta |
| AZ3a_ORF_21145 | AZ14_ORF_09665 |  | hypothetical protein |
| AZ3a_ORF_21150 | AZ14_ORF_09670 |  | hypothetical protein |
| AZ3a_ORF_21155 | AZ14_ORF_09675 |  | hypothetical protein |
| AZ3a_ORF_21160 | AZ14_ORF_09680 |  | hypothetical protein |
| AZ3a_ORF_21165 | AZ14_ORF_09685 |  | hypothetical protein |
| AZ3a_ORF_21175 | AZ14_ORF_09695 |  | hypothetical protein |
| AZ3a_ORF_21180 | AZ14_ORF_09700 |  | cell division protein DivIVA |
| AZ3a_ORF_21185 | AZ14_ORF_09705 |  | hypothetical protein |
| AZ3a_ORF_21200 | AZ14_ORF_05885 |  | hypothetical protein |
| AZ3a_ORF_21205 | AZ14_ORF_05880 |  | XRE family transcriptional regulator |
| AZ3a_ORF_21210 | AZ14_ORF_05860 |  | Zn-dependent protease |
| AZ3a_ORF_21750 | AZ14_ORF_08755 | *ulaG* | L-ascorbate-6-phosphate lactonase |
| AZ3a_ORF_21755 | AZ14_ORF_08760 | *ulaR* | transcriptional antiterminator |
| AZ3a_ORF_21760 | AZ14_ORF_08765 | *sgaESgbE* | ribulose phosphate epimerase |
| AZ3a_ORF_21765 | AZ14_ORF_08770 | *sgaU* | xylulose 5-phosphate 3-epimerase |
| AZ3a_ORF_21770 | AZ14_ORF_08775 | *sgaH* | 3-keto-L-gulonate-6-phosphate decarboxylase |
| AZ3a_ORF_21775 | AZ14_ORF_08780 | *ulaA* | PTS ascorbate transporter subunit IIA |
| AZ3a_ORF_21780 | AZ14_ORF_08785 | *ulaB* | PTS ascorbate transporter subunit IIB |
| AZ3a_ORF_21785 | AZ14_ORF_08790 | *ulaC* | PTS ascorbate transporter subunit IIC |
| AZ3a_ORF_22175 | AZ14_ORF_09660 |  | hypothetical protein |
| AZ3a_ORF_22190 | AZ14_ORF_09645 |  | hypothetical protein |
| AZ3a_ORF_22210 | AZ14_ORF_09620 |  | Toxin PezT |
| AZ3a_ORF_22215 | AZ14_ORF_09615 |  | hypothetical protein |
| AZ3a_ORF_22220 | AZ14_ORF_09610 |  | ATPase |
| AZ3a_ORF_22225 | AZ14_ORF_09605 |  | Chemotaxis protein |
| AZ3a_ORF_22230 | AZ14_ORF_09600 |  | hypothetical protein |
| AZ3a_ORF_22235 | AZ14_ORF_09595 |  | DNA primase |
